# Efficacy and Safety of Docetaxel in Elderly Patients With Metastatic Castration-Resistant Prostate Cancer

**DOI:** 10.1200/JGO.2016.007807

**Published:** 2017-03-27

**Authors:** Manuel Caitano Maia, Allan A. Lima Pereira, Liana Valente Lage, Natalia Moreno Fraile, Victor Van Vaisberg, Guilherme Kudo, Romualdo Barroso-Sousa, Diogo Assed Bastos, Carlos Dzik

**Affiliations:** **All authors:** Universidade de São Paulo, São Paulo, Brazil.

## Abstract

**Purpose:**

Limited data are available about the tolerability and clinical outcomes of elderly patients with metastatic castration-resistant prostate cancer (mCRPC) who are treated with docetaxel. We evaluated the efficacy and safety of docetaxel as first-line chemotherapy for patients with mCRPC who were treated in our institution.

**Materials and Methods:**

We retrospectively identified patients with mCRPC and a Karnosfky performance status of 60% or greater treated with docetaxel on any schedule as first-line chemotherapy between 2008 and 2013. The primary end point was a comparison of median overall survival (OS) according to age in this population. Secondary end points were comparisons of the rates of severe toxicities, prostate-specific antigen (PSA) decline of 50% or greater, and time to progression (TTP). Results were stratified by three age groups: younger than 65 years, 65 to 74 years, and 75 years or older.

**Results:**

Among the 197 patients included, 68 (34%) were younger than 65 years, 85 (43%) were 65 to 74 years, and 44 (22%) were 75 years or older. The mean number of comorbidities was not different among groups (1.19 *v* 1.32 *v* 1.43; *P* = .54). Patients younger than 65 years received a higher cumulative dose of docetaxel (450 mg/m^2^ *v* 382 mg/m^2^ *v* 300 mg/m^2^; *P* = .004). The rates of PSA decline of 50% or greater (41% *v* 47% *v* 36.4%; *P* = .51) and the median TTP (5.13 *v* 5.13 *v* 4.7 months; *P* = .15) were comparable among all groups. The median OS was longer in the group of patients younger than age 65 years (19.6 *v* 12.4 *v* 12.3 months; *P* = .012). Rates of any grade 3 or higher adverse event were not different among groups (63.2% *v* 71.8% *v* 54.5%; *P* = .14).

**Conclusion:**

Administration of docetaxel in elderly patients who had good performance status was well tolerated. Rates of PSA decline and TTP were similar to those of younger patients, but median survival was lower.

## INTRODUCTION

Prostate cancer is the second leading cause of cancer-related death in men and is a major health problem worldwide, with estimated more than 220,000 new occurrences in the United States in 2015.^1^ The main known risk factor related to prostate cancer is age: roughly 62% of new occurrences worldwide are diagnosed in men older than 65 years.^2^

The standard initial treatment of metastatic prostate cancer since the 1940s has been androgen-deprivation therapy (ADT), which usually leads to disease control for approximately 18 to 24 months in the setting of castration levels of testosterone.^3^ Recently, however, it was shown that docetaxel added to ADT for patients with metastatic castration-sensitive disease significantly increases survival, especially in patients with high-volume disease.^4-6^ Although many nonchemotherapy agents have emerged as new treatment options for these patients, most patients with metastatic castration-resistant prostate cancer (mCRPC) at some point will be candidates for chemotherapy to improve symptoms related to progressive disease. Docetaxel was the first chemotherapy agent to demonstrate an overall survival (OS) benefit in this scenario.^7,8^

Chemotherapy safety and tolerability, however, are concerns, because most patients are elderly and many have comorbidities.^8^ Limited evidence exists to guide treatment decisions in older patients. Some international societies have published clinical guidelines to facilitate patient selection and treatment approach.^9^ However, they are not routinely used in clinical practice.

The aim of this study was to evaluate the efficacy, safety, and toxicities attributable to docetaxel in the elderly population compared with younger patients with mCRPC who were treated at our center. We hypothesized that older patients might experience worse OS and a poorer safety profile than younger patients in this retrospective analysis.

## MATERIALS AND METHODS

### Patients

This study is a retrospective analysis of patients with mCRPC treated at Instituto do Cancer do Estado de São Paulo, Brazil. We included in this analysis patients who initiated docetaxel as first-line chemotherapy at our institution between June 2008 and October 2013 with the following eligibility criteria: (1) disease progression in the setting of surgical or chemical castration on the basis of an increasing PSA (defined as two consecutive increases in PSA value at least 2 weeks apart from each other) or radiographic evidence of disease progression in soft tissue or bone with or without disease progression on the basis of the PSA value or symptoms attributable to prostate cancer metastasis; (2) Karnofsky performance status (KPS) of 60% or greater; and (3) adequate bone marrow function (hemoglobin > 8.5 g/dL; absolute neutrophil count > 1,000/mm^3^; platelet count > 100,000/mm^3^). There was no upper age limit for inclusion. We excluded patients for the following reasons: (1) treatment in study protocols; (2) poor performance status (KPS < 60%); (3) delivery of initial chemotherapy cycle as inpatient or at another institution; or (4) receipt of any other chemotherapy agents before docetaxel. Comorbidity data were obtained from patient charts, as documented by the attending physician. Institutional review board and ethics committee approvals were given to conduct this retrospective analysis.

### Treatment, Assessment, and Outcomes

Patients—including those patients who started with a standard dose or a reduced dose because of older age, performance status, or other factors—received treatment schedules and doses according to physician choice. Also, some patients who started with an alternative lower dose went on to receive the full dose after first or second cycles if well tolerated. Treatment was maintained until progressive disease occurred; progressive disease was defined as worsening symptoms, PSA increase of more than 25% above the nadir, new radiologic lesions, increase in lesion size, maximum treatment benefit, or treatment-limiting toxicity.

The primary end point was OS, which was defined as the time from the start of therapy (docetaxel) to death as a result of any cause. Secondary end points were rate of PSA decline of 50% or greater; rate of grade 3 or greater adverse events (AEs); and time to progression (TTP), defined as the time from docetaxel initiation to progressive disease. Stable disease according to PSA values was defined as a PSA decline of less than 30% or a PSA increase of no more than 25% above the nadir. Progressive disease according to PSA values was defined as a PSA increase of greater than 25% above the nadir. Progressive disease also was considered in patients who had documented new sites of disease or worsening bone pain.

Safety outcomes included the numbers and proportion of patients who experienced AEs of grade 3 or higher. We retrospectively assessed AEs and assigned grade levels on the basis of the National Cancer Institute Common Terminology Criteria for Adverse Events (CTCAE), version 3.0.

All patients without previous orchiectomy received continuous luteinizing hormone–releasing hormone agonist (goserelin) every 3 months and were monitored for castration levels of testosterone. Patients underwent clinical and laboratory evaluation, which included complete blood cell counts, blood chemistry, and PSA levels; evaluation usually occurred before the next chemotherapy cycle (every 3 weeks).

The endocrine therapies available for use within our institution consisted of bicalutamide, flutamide, diethylstilbestrol, ketoconazole, dexamethasone, or prednisone. None of the patients included in this analysis received abiraterone or enzalutamide before or after docetaxel chemotherapy, because these drugs were not available at our center for routine use during the study period.

### Statistics

Baseline demographics and clinical characteristics were summarized with descriptive statistics. Our analysis was based on three age groups: younger than 65 years, 65 to 74 years, and 75 years or older. Age groups were chosen on the basis of commonly used age strata in published literature. PSA responses and AEs were reported as relative rates. The categoric parameters were compared with the two-sided Pearson χ^2^ test or Fisher's exact test, as appropriate. Continuous variables were analyzed by applying the analysis of variance for comparison of normally distributed variables and the Kruskal-Wallis test for non-normally distributed ones. Time-to-event variables were calculated from the start of therapy with docetaxel according to the Kaplan-Meier method and were compared by means of the log-rank test. We used Cox proportional hazard regression models to estimate hazard ratios and to investigate whether the effect of age group was modified by adjustments for the following covariates: site of metastasis, best PSA response, Gleason score, KPS, comorbidities, and initial chemotherapy dose. We calculated hazard ratios and 95% CIs for OS and TTP with Cox proportional hazards regression. All tests were two sided, and a *P* value of less than .05 was considered statistically significant. SPSS software (version 20.0; SPSS, Chicago, IL) was used for statistical analyses.

## RESULTS

### Patients

**Fig 1:**
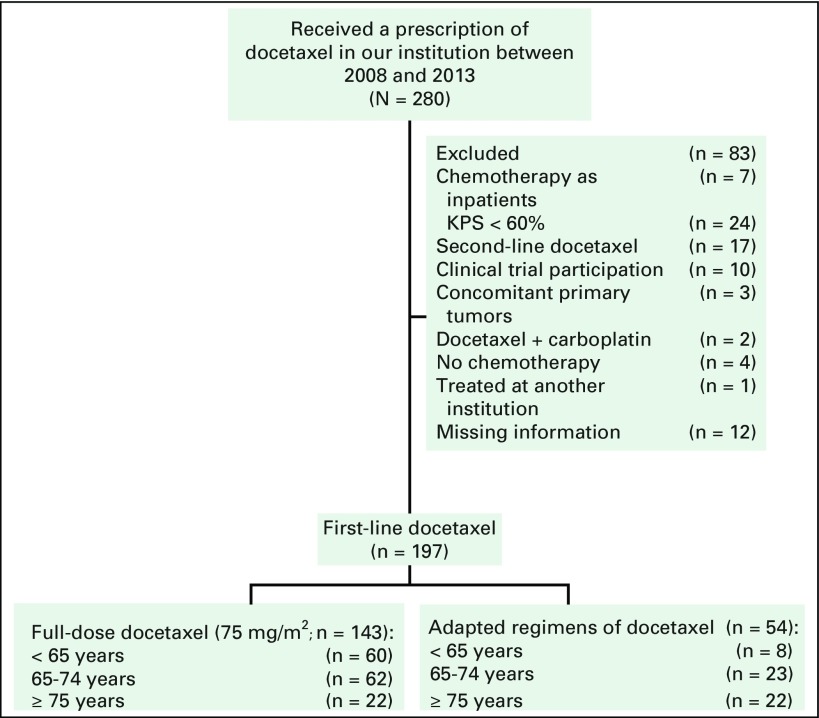
Flow chart. A total of 280 patients were identified initially; 83 were excluded on the basis of exclusion criteria (inpatients when received chemotherapy [n = 7]; Eastern Cooperative Oncology Group performance status > 2 [n = 24]; received a previous chemotherapy regimen [n = 17]; received docetaxel as part of a study protocol [n = 10]; another concomitant primary tumor [n = 3]; received docetaxel combined with carboplatin [n = 2]; did not receive any chemotherapy [n = 4]; received the treatment at another institution [n = 1]; or did not have enough information on charts [n = 12]). KPS, Karnofsky performance status.

Between June 2008 and October 2013, among all patients who received docetaxel at our institution for prostate cancer, 197 men fulfilled the criteria for this analysis (Fig 1). In the overall population, 68 men (34%) were younger than 65 years, 85 (43%) were age 65 to 74 years, and 44 (22%) were 75 years or older. The median age was 70 years. The majority of patients (72%) had a KPS of 80% to 100%. Only 22% of patients younger than age 65 years had a KPS of 60% to 70% compared with 32.9% of those who were age 65 to 74 years and 25% of those who were 75 years or older (*P* = .29). Patient demographic and clinical characteristics are listed in Table 1.

**Table 1:**
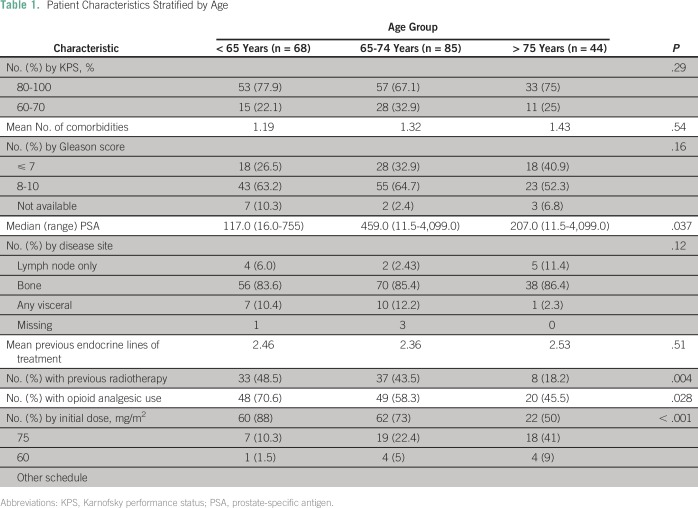
Patient Characteristics Stratified by Age

There was a trend toward a higher number of comorbidities in those age 75 years or older compared with those age 65 to 74 years and with those younger than 65 years (mean of number of comorbidities, 1.19 *v* 1.32 *v* 1.43, respectively; *P* = .54). The majority of patients in the study had bone disease (n = 164; 83%), and its occurrence was well balanced across all age groups (83.6% *v* 85.4% *v* 86.4% for groups younger than 65 years, 65 to 74 years, and 75 years or older, respectively; *P* = .12). There was a trend toward a lower incidence of visceral metastasis in the older group (75 years or older), which was not statistically significant (10.4% *v* 12.2% *v* 2.3% for groups younger than 65 years, 65 to 74 years, and 75 years or older, respectively; *P* = .12).

Patients age 65 to 74 years had higher PSA values at baseline than patients in other age groups (117 *v* 459 *v* 207 ng/mL for groups younger than 65 years, 65 to 74 years, and 75 years or older, respectively; *P* = .037). Patients 75 years or older received less palliative radiotherapy (48.5% *v* 43.5% *v* 18.2% for groups younger than 65 years, 65 to 74 years, and 75 years or older, respectively; *P* = .004), and patients younger than 65 years were more likely to use opioid drugs (70% *v* 58.3% *v* 45.5% for groups younger than 65 years, 65 to 74 years, and 75 years or older, respectively; *P* = .028) before initiation of docetaxel. In the study population, 173 patients (87%) used at least two hormonal lines of treatment, and at least 91 patients (46%) used three or more endocrine therapies. There was no difference in the mean number of previous endocrine lines used before docetaxel among the three age groups (*P* = .51; Table 1).

### Treatment Patterns

In general, most patients (73%) started treatment with docetaxel 75 mg/m^2^ on an every-3-week schedule. However, only 50% of patients age 75 years or older started with the standard dose, whereas 73% of patients age 65 to 74 years and 88% of patients younger than 65 years initiated with the full dose. The proportions of conversion from the full dose (ie, 75 mg/m^2^) to alternative regimens were 28.3%, 27.4%, and 50% for groups younger than 65 years, 65 to 74 years, and 75 years or older, respectively (*P* = .12; Appendix Table A1). The median number of cycles per patient among all groups was six (range, one to 13 cycles). The cumulative dose, however, was different among groups. Patients younger than 65 years had a higher median cumulative dose than others (450 mg/m^2^ *v* 382 mg/m^2^ *v* 300 mg/m^2^ for groups younger than 65 years, 65 to 74 years, and 75 years or older, respectively; *P* = .004).

Among all groups, the main reason for treatment discontinuation was disease progression (45%), followed by toxicity (28%) and treatment completion (18%). Although older patients (75 years or older) discontinued treatment because of toxicity more often than patients in other groups (36.4% *v* 33% *v* 19% for groups age 75 years or older, 65 to 74 years, and younger than 65 years, respectively), this difference was not statistically significant (*P* = .32).

### Efficacy

At the time of analysis (April 2015), 190 patients had discontinued treatment, 140 had died, and three were lost to follow-up. The median follow-up time after treatment initiation was 14.6 months.

**Fig 2:**
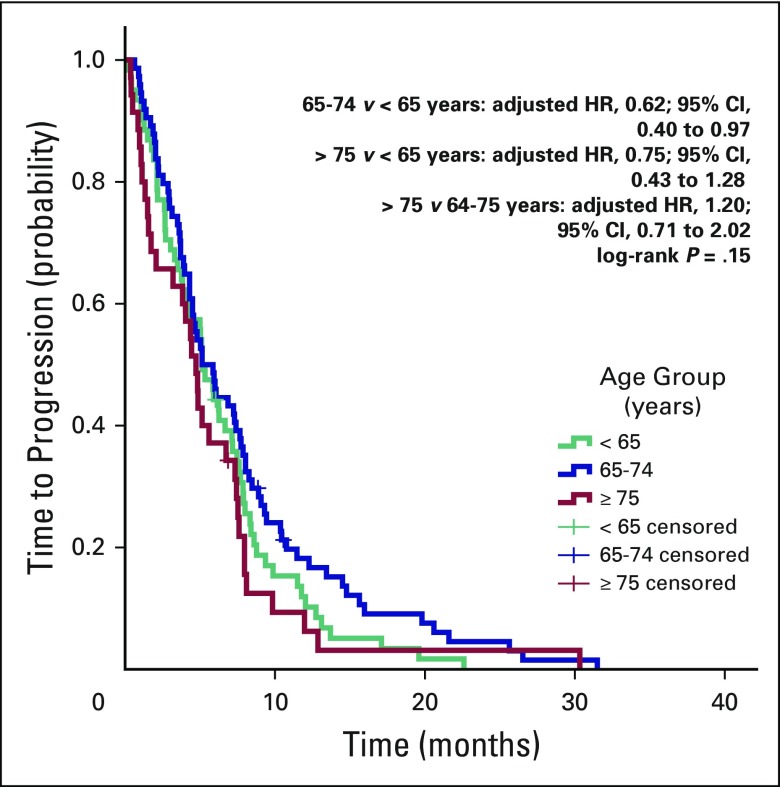
Time-to-treatment progression according to age group. HR, hazard ratio.

The rates of PSA decrease of 50% or greater were 41%, 47%, and 36.4% for groups younger than 65 years, 65 to 74 years, and 75 years or older, respectively (*P* = .51). The median TTP was 5.13 months (range, 4.14 to 6.1 months) for patients younger than 65 years, 5.13 months (range, 3.6 to 6.6 months) for patients age 65 to 74 years, and 4.7 months (range, 3.7 to 5.7 months) for patients age 75 years or older. Results of age-group comparisons were as follows: for 65 to 74 years versus younger than 65 years, the adjusted hazard ratio (HR) was 0.62 (95% CI, 0.40 to 0.97); for 75 years and older versus younger than 65 years, the adjusted HR was 0.75 (95% CI, 0.43 to 1.28); and for 75 years and older versus 65 to 74 years, the adjusted HR was 1.20 (95% CI, 0.71 to 2.02; log-rank *P* = .15; Fig 2).

**Fig 3:**
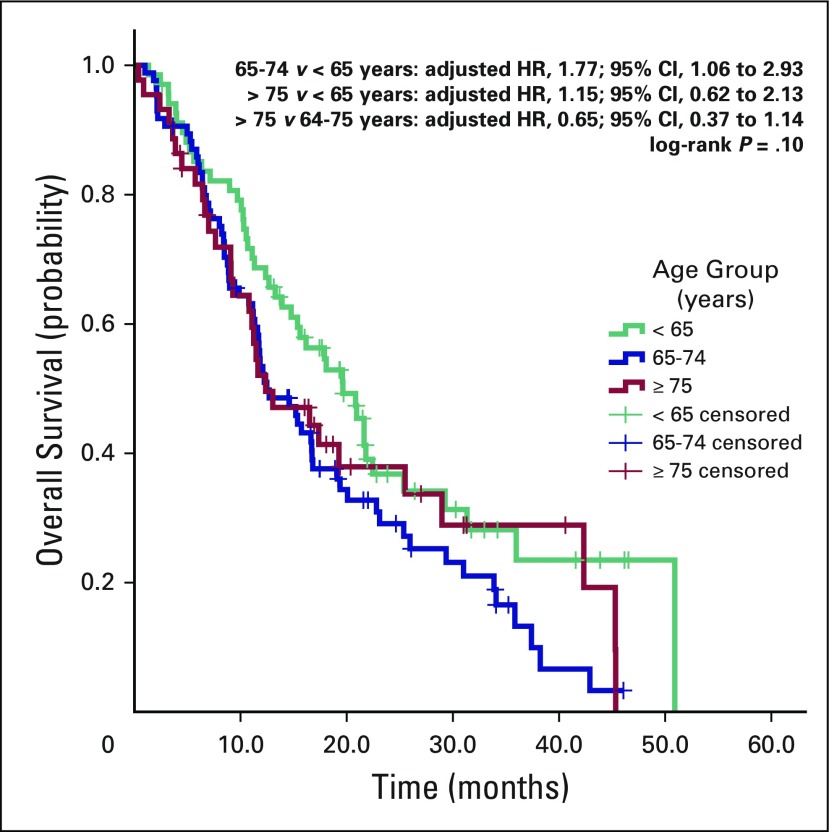
Kaplan-Meier curves for overall survival according to age groups. HR, hazard ratio.

**Fig A1:**
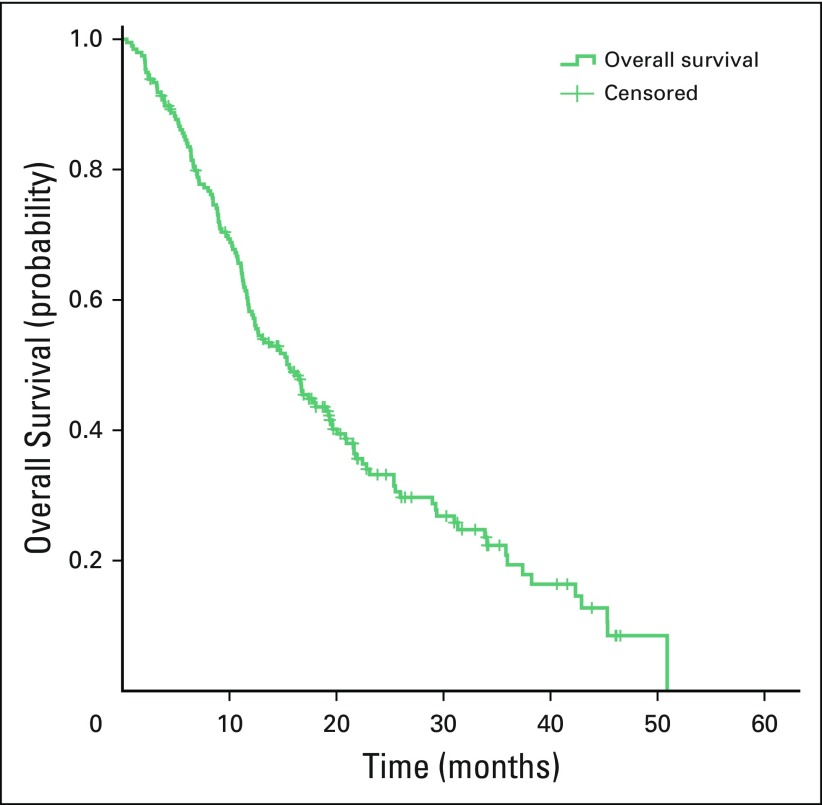
Kaplan-Meier curve for overall survival of the whole cohort (15.6 months).

The median OS times were 19.6 months (range, 15.1 to 24.1 months), 12.4 months (range, 9.0 to 15.9 months), and 12.3 months (range, 6.0 to 18.0 months) for groups younger than 65 years, 65 to 74 years, and 75 years or older (*P* = .012). The median OS for the whole cohort was 15.6 months (Appendix Fig A1). Results of age-group comparisons were as follows: for 65 to 74 years versus younger than 65 years, the adjusted HR was 1.77 (95% CI, 1.06 to 2.93); for 75 years and older versus younger than 65 years, the adjusted HR was 1.15 (95% CI, 0.62 to 2.13); and for 75 years and older versus 65 to 74 years, the adjusted HR was 0.65 (95% CI, 0.37 to 1.14; log-rank *P* = .10; Fig 3).

### Toxicity

AEs grades 3 or 4 were common across all age groups, mostly as a result of hematologic toxicities. Only grade 3 or greater AEs were reported (Table 2). Although those patients age 75 years or older developed grade 3 or greater AEs less often in general (54.5% *v* 71.8 *v* 63.8 for ages 75 years or older, 65 to 74 years, or younger than 65 years, respectively), this difference was not statistically significant (*P* = .14). However, significant differences were observed for specific toxicities. Patients age 75 years or older developed anemia (17.6 *v* 22.4 *v* 11.4% for groups younger than 65 years, 65 to 74 years, and 75 years or older, respectively; *P* = .008), neutropenia (48.5% *v* 56.5% *v* 25.0% for groups younger than 65 years, 65 to 74 years, and 75 years or older, respectively; *P* = .011), and febrile neutropenia (5.9% *v* 15.3% *v* 2.3% for groups younger than 65 years, 65 to 74 years, and 75 years or older, respectively; *P* = .034) less often. All other AEs were not significantly different among age groups.

**Table 2:**
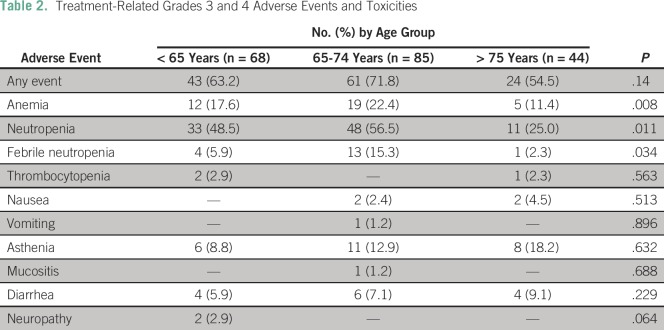
Treatment-Related Grades 3 and 4 Adverse Events and Toxicities

## DISCUSSION

Because many patients with mCRPC are often older, the importance of age on tolerability and efficacy of chemotherapy-based treatment is of major concern.^10^ To date, there is scarce evidence on which to base treatment decisions for older patients with prostate cancer, because this group is underrepresented in clinical trials.^11,12^ Furthermore, the heterogeneity of the aging process contributes to the complexity of treatment decisions.^10^ In clinical practice, treatment decisions often are made on the basis of chronologic age. Thus, we have an unmet need to better select appropriate treatment for elderly or frail patients.^13^

Since 2004, docetaxel-based treatment has been shown to improve survival compared with other regimens and has become the standard first-line chemotherapy in mCRPC.^7,8^ In the TAX 327 trial, docetaxel given in an every-3-week schedule was associated with better survival than weekly administration of either docetaxel or mitoxantrone. In this trial, both every-3-week and weekly docetaxel schedules were significantly associated with improvement in quality of life and PSA response, but increased OS was seen only in the every-3-week schedule.^8^ In the Southwest Oncology Group trial, which compared docetaxel and estramustine with mitoxantrone, docetaxel was associated with increases in OS, progression-free survival, and rates of PSA decline.^7^

Even in fit elderly men, docetaxel administered every 3 weeks is the preferable treatment option, because weekly administration was not associated with increased survival and did not have better tolerance according to the results of several studies, which also demonstrated that the improvement in OS was independent of age.^8,10,14^ In the TAX 327 trial, weekly docetaxel showed the same rates of grade 3 and 4 toxicities. Italiano et al^15^ showed that the weekly schedule was poorly tolerated by frail patients. However, independent of the schedule used, evidence suggests that older men (age 75 years or older) and those who received previous radiotherapy for localized disease are more prone to have adverse events^16^ and therefore should be monitored closely for toxicity.^10,14,17^

In this study, except for the median baseline PSA value, the previous use of radiotherapy, and opioid use before chemotherapy initiation, older patients did not differ significantly from other age groups in baseline characteristics, such as performance status, disease burden, and even the number of comorbidities. These data only confirm the usual selection of therapies made on the basis of physician perceptions about patient tolerability, especially when therapies are administered outside clinical trials, which often is the case for patients older than age 75 years.

Despite baseline characteristics similar to those of other age groups, only 50% of older patients received standard docetaxel doses (75 mg/m^2^) at initiation of treatment. This probably was due to the perception of frailty of elderly patients by the treating physicians, although the specific reasons for dose reduction were not reported in patient charts. It is also possible that adapted regimens were chosen according to physician discretion, which again reflected physician worries about patient tolerance; this may have been based solely on chronologic age.

Geriatric risk assessment is a crucial step to select fit older patients to receive chemotherapy.^17,18^ The International Society of Geriatric Oncology issued a guideline to recommend treatment according to general health status but not age.^17^ Elderly patients should be given the same treatment options as their younger counterparts if they are clinically fit.^9^ Those with reversible impairments should be compensated before appropriate treatment, whereas those with irreversible impairments (the really frail ones), should receive adapted options or best supportive care.^9,17,19^

Although the OS analysis was adjusted for the initial chemotherapy dose schedule, OS significantly differed among age groups: patients younger than 65 years had better outcomes than patients in other age groups (ie, 65 to 74 years and 75 years or older). This contrasts with results from other trials that evaluated the efficacy of docetaxel in patients age 75 years or older compared with younger patients. In a small, randomized trial comparing docetaxel in younger and older patients, there were no differences in survival or PSA response,^20^ although the sample size was small (N = 51). In the TAX 327 trial and the Southwest Oncology Group trial, OS improvements were present irrespective of age.^7,8,14^ A pooled analysis of two phase II trials to evaluate weekly docetaxel in patients age 70 years or older compared with younger patients showed equivalent results.^21^ Our results, however, are consistent with those reported in a retrospective analysis of patients with mCRPC who were treated in routine clinical practice with every-3-week docetaxel (the median survival was 13.6 months)^22^ and with the OS reported by Veccia et al^18^ (ie, 14 months in patients age 80 years or older treated with first-line docetaxel who did not have access to newer drugs, such as abiraterone, enzalutamide, and cabazitaxel). Thus, our findings about OS could not be attributed to a lower-than-expected survival in older patients. Another explanation for the lower survival is the lower cumulative dose of docetaxel received by older patients.

In routine practice, the elderly population usually receives weekly docetaxel with the premise that it is better tolerated than an every-3-week schedule.^10,13^ Although there was a small difference in toxicities in older men treated with weekly versus every-3-week docetaxel in the TAX-327 trial,^10,14^ other trials suggest that the weekly regimen is less safe than believed, mostly because nonhematologic toxicity, such as diarrhea and fatigue, often leads to treatment discontinuation.^15,23^ However, the data from these studies should be interpreted with caution, because most patients who received the weekly schedule had a worse general health status.^15^ Conversely, as recently reported by Kellokumpu-Lehtinen et al,^24^ docetaxel administered on an every-2-weeks schedule with a dose of 50 mg/m^2^ was associated with a significantly lower rate of grades 3 and 4 AEs, so the twice-weekly schedule would be deemed more appropriate for those less likely to tolerate the standard doses, such as those in the elderly population.^24^ Our results show a high incidence of grades 3 and 4 hematologic toxicities. This could be because patients were not treated in a clinical trial, so the timing of laboratory analysis was not standardized, and many patients may have underwent blood collection during the nadir of chemotherapy instead of right before the next cycle. Our results also show that older patients had statistically significant less anemia, neutropenia, and febrile neutropenia. This could be explained by the lower cumulative dose they received, which reflects either a lower tolerability or the more frequent use of adapted regimens, such as weekly docetaxel. More important, however, is that the rates of febrile neutropenia encountered in our study (5.9% *v* 15.3% *v* 2.3% in age groups younger than 65 years, 65 to 74 years, and 75 years or older, respectively) were comparable to those reported by other authors in patients not treated in clinical trials (10%).^22^

Interestingly, although older patients (75 years or older) discontinued treatment because of toxicity more often than patients in other groups, this difference was not statistically significant. The lack of statistical significance may be a result, in part, of lower doses received by older adults and of the small sample size.

This study has inherent limitations because of its retrospective nature. It included a small number of patients older than 75 years old (n = 44), and only 50% of them received full-dose chemotherapy. Although the number represents real-life data, this small sample precludes definitive conclusions for this subgroup. Also, clinical data were extracted from clinical notes, so this may have led to underdetection of comorbidities, rates of toxicities, and accurate performance statuses. Therefore, conclusions from this analysis should be made with caution.

Another interesting point for speculation is how the newer androgen receptor–directed therapies, such as abiraterone and enzalutamide, could have interfered with results about survival or toxicities in the elderly population with mCRPC. Unfortunately, no hypothesis can be made about the effect, simply because those drugs are not available in our public system yet.

In summary, our results suggest that, in the real-world setting, docetaxel continues to be a reasonable option, which has a good safety profile, in the elderly population, even if survival may be compromised by dose reduction. This compromise is not necessarily a negative point, because quality of life at the age of 75 years or older is as important as survival. We should continue to follow the guidelines to better identify the fit elderly patients who, therefore, can receive full doses to accomplish the best results, as suggested by evidence-based medicine. For those unfit patients, finding the best chemotherapeutic options continues to be an unmet need.

## Figures and Tables

**Table A1:**
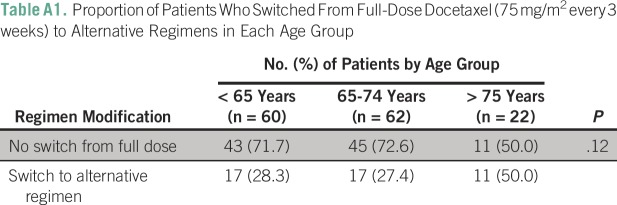
Proportion of Patients Who Switched From Full-Dose Docetaxel (75 mg/m^2^ every 3 weeks) to Alternative Regimens in Each Age Group
